# Parathyroid hormone in renal transplanted recipients; a single center study

**Published:** 2013-01-01

**Authors:** Hamid Nasri, Mahmoud Rafieian-Kopaei

**Affiliations:** ^1^Department of Internal Medicine, Shahrekord University of Medical Sciences, Iran; ^2^Medical Plants Research Center, Shahrekord University of Medical Sciences, Shahrekord, Iran

**Keywords:** Parathormone, Transplantation, Parathyroid gland

## Abstract

This investigation, aimed to study of intact parathormone (iPTH) and calcium (Ca) in a group of kidney transplanted patients and also we aimed to test the relationship of iPTH with various demographic data of kidney transplanted recipients. We studied 72 kidney transplanted persons with mean ages of 44±12 years. In this study, mean iPTH was 18.4±8.2 Pg/mL (median=16.5). A negative correlation of iPTH with creatinine clearance (r=-0.44, p<0.001) was seen. There was no any association between the time of kidney transplantation and serum iPTH (p>0.05). In contrast to previous findings, in our patients, there was not secondary hyperparathyroidism. The results revealed suppressed PTH secretion. The reason may be due to excessive intake of calcium and Vitamin D analogues, which may suppress parathyroid hormone secretion.

**Implication for health policy/practice/research/medical education:**

In a study on 72 kidney-transplanted persons with mean ages of 44±12 years. The mean intact PTH was 18.4±8.2 Pg/mL (median=16.5). A negative correlation of intact PTH with creatinine clearance (r=-0.44, p<0.001) was seen. There was no any association between the time of kidney transplantation and serum iPTH (p>0.05). In contrast to previous publications, in our patients, there was not secondary hyperparathyroidism. The results revealed suppressed PTH secretion. The reason may be due to excessive intake of calcium and vitamin D analogues, which may suppress parathyroid hormone secretion.

Kidney Transplantation (KTx) is a treatment of choice for patients with end-stage renal disease (1,2). KTx improves most of the metabolic disturbs (1). Post-transplant bone disease is a heterogeneous problem due to various factors, consisting gender, age, presence of hyperparathyroidism, corticosteroids and treatment with calcineurin inhibitors (1,2). Indeed, it is essential to test parathyroid gland function as a regular follow up of these patients. In this study, we aimed to consider the post-transplant function of parathyroid gland in a group of KTx patients.

## Patients and Methods

### Patients

This cross-sectional study was performed on a group of renal transplanted recipients. Exclusion criteria were presence of acute rejection, infection, antibiotic taking during the past two months. The immunosuppressive protocol of the patients contained of a combination of prednisolone 7.5 mg/d for all of the patients, cyclosporine at a mean dosage of 190±60 mg/d, and mycophenolate mofetile in 46% of the patients at a dosage of 1500 ±500 mg/d /or azathioprine at a dosage of 50 to 100 mg/d in the rest of the patients.

### Laboratory methods

Serum intact parathormone (iPTH) levels were measured after an overnight fast. iPTH was measured by Radioimmunoassay method (normal range of values: 10-65 pg/mL). Blood samples were collected also for biochemical analysis including serum creatinine, blood urea nitrogen (BUN), levels, using standard kits. Creatinine clearance was assessed from serum creatinine, age and body weight (3).

### Ethical approval

All patients signed the consent form for participation in this study. Research study was approved by the ethics committee of Shahrekord University of Medical Sciences, Iran.

### Statistical Analysis

Descriptive results were expressed as the mean±SD and median values. For correlations, partial correlation test was used. Comparisons between groups were done using student’s t-test. For normalization of the iPTH data, their second square values were used. All statistical analysis were performed with the SPSS 12 (SPSS Inc., Chicago, USA). Statistical significance was determined at a p<0.05.

## Results

A total of 72 patients were included to this study (47 male, 25 female). The mean patients’ age was 44±12 years. The mean time of KTx was 67.5±42 months Mean serum iPTH was 18.4±8.2 Pg/mL. In this study an inverse association of serum iPTH with creatinine clearance (r=-0.44, p<0.001) was found. No significant association between the duration KTx and serum iPTH (p>0.05) was seen.

## Discussion

In the present study, we found an inverse association of iPTH with creatinine clearance. There was not any significant association between the duration of KTx and iPTH. Our patients’ mean intact parathormone was 18.4 (±8.2) pg/mL, which referred to the suppressed level of parathormone. The cause may be due to excessive intake of vitamin D and calcium analogues. In fact, secondary hyperparathyroidism is a common complication in patients with chronic kidney disease (4–8). Renal transplantation improves the abnormalities responsible for secondary hyperparathyroidism in the first months (8–10). Nevertheless, elevated iPTH level has been found in >25% of patients one year after renal transplantation in the presence of good kidney function (8–11).

## Conclusion

We concluded that, the reason of suppressed level of parathormone in our patients may be due to excessive intake of vitamin D and calcium analogues, which suppress the parathyroid gland secretion, and may susceptible these patients to a dynamic bone disease.

## Authors’ contributions

HN and MRK wrote the manuscript equally.

## Conflict of interests

The authors declared no competing interests.

## Ethical considerations

Ethical issues (including plagiarism, misconduct, data fabrication, falsification, double publication or submission, redundancy) have been completely observed by the authors.

## Funding/Support

This study was supported by a grant from Shahrekord University of Medical Sciences, Iran. This study extracted from M.D thesis.
